# An Extended EPQ-Based Problem with a Discontinuous Delivery Policy, Scrap Rate, and Random Breakdown

**DOI:** 10.1155/2015/621978

**Published:** 2015-03-04

**Authors:** Singa Wang Chiu, Hong-Dar Lin, Ming-Syuan Song, Hsin-Mei Chen, Yuan-Shyi P. Chiu

**Affiliations:** ^1^Department of Business Administration, Chaoyang University of Technology, Taichung 413, Taiwan; ^2^Department of Industrial Engineering & Management, Chaoyang University of Technology, Wufong District, Taichung 413, Taiwan

## Abstract

In real supply chain environments, the discontinuous multidelivery policy is often used when finished products need to be transported to retailers or customers outside the production units. To address this real-life production-shipment situation, this study extends recent work using an economic production quantity- (EPQ-) based inventory model with a continuous inventory issuing policy, defective items, and machine breakdown by incorporating a multiple delivery policy into the model to replace the continuous policy and investigates the effect on the optimal run time decision for this specific EPQ model. Next, we further expand the scope of the problem to combine the retailer's stock holding cost into our study. This enhanced EPQ-based model can be used to reflect the situation found in contemporary manufacturing firms in which finished products are delivered to the producer's own retail stores and stocked there for sale. A second model is developed and studied. With the help of mathematical modeling and optimization techniques, the optimal run times that minimize the expected total system costs comprising costs incurred in production units, transportation, and retail stores are derived, for both models. Numerical examples are provided to demonstrate the applicability of our research results.

## 1. Introduction

This paper focuses on optimizing a producer-retailer integrated economic production quantity- (EPQ-) based problem with a discontinuous delivery policy, scrap rate, and random breakdown. The EPQ model was first introduced by Taft [1] and its concept has since been frequently implemented by manufacturing firms to determine the most economic replenishment batch sizes for the products that need to be produced in-house [2, 3]. Although the traditional EPQ model assumes a perfect condition in each production run, in real manufacturing environments due to process deterioration or other uncontrollable factors generation of defective items and random breakdown are inevitable [4]. Widmer and Solot [5] applied a queuing network theory to the study of a breakdown and maintenance operation problem. A simple way of modeling these perturbations was proposed to take into account the performances evaluation of the flexible manufacturing system (FMS) (including the production rate and machine utilization). The analytical and simulation results were compared in order to demonstrate the accuracy of their modeling technique. Yu and Bricker [6] presented an informative application of Markov's chain analysis to a multistage manufacturing problem. They also pointed out an error in the literature that had been undetected for many years. Groenevelt et al. [7] investigated the effects of breakdowns and corrective maintenance on the economic batch sizing decisions. Two inventory control policies were examined in the case of a breakdown, namely, the no-resumption (NR) and abort-resume (AR). The NR control policy assumes that after a breakdown situation is handled the production of the interrupted lots is not resumed while the AR control policy assumes that if the current on-hand inventory is below a certain threshold level, then the production is immediately resumed after a breakdown situation is taken care of. Their research results indicated that this control structure is optimal among all stationary policies and offered the exact optimal and closed form approximate lot sizing formulas and bounds on average cost per unit time for the approximations. Widyadana and Wee [8] developed deteriorating items production inventory models with random breakdown and stochastic machine repair time. The repair time is assumed to be independent of the breakdown rate. They applied the classical optimization technique to the problem and derived an optimal solution. Through a numerical example and sensitivity analysis, they showed that the production and demand rates are the most sensitive parameters to the optimal uptime, and the demand rate is the most sensitive variable to the system costs for the stochastic model with exponential distribution repair time. Chiu et al. [9] determined the optimal replenishment run time for an EPQ-based inventory model with nonconforming items and breakdown. Their model assumes that after a Poisson distributed breakdown occurs, the machine goes under repair instantly and the production of the interrupted lot resumes immediately when the machine is fixed and restored. The system also considers a uniformly distributed scrap rate associated with the production process. A mathematical model along with a recursive searching algorithm is used in their study to derive the optimal replenishment policy that minimizes the total system costs. A numerical example was provided to demonstrate the practical application and better cost efficiency of the proposed policy compared to a breakdown that occurs under a no-resumption policy. Additional studies relating to the issues of product quality, unreliable production equipment, and their consequence quality assurance can be found in [10–17].

Unlike the assumption of a continuous inventory issuing policy of the traditional EPQ model, in real supply chain environments, the discontinuous multidelivery policy is often used when finished products need to be transported to retailers or customers outside the production units. Schwarz et al. [18] determined the fill-rate of a one-warehouse, N-identical retailer distribution system. An approximation model was adopted from a prior work to maximize the system fill-rate subjected to a constraint on safety stock. The properties of the fill-rate policy were used to provide managerial insights into system optimization. Sarker and Khan [19] examined a manufacturing system that procures raw materials in a lot from suppliers and processes them into the finished products that are subsequently shipped to outside customers at fixed points in time. The system cost function for the model was formulated by including both raw materials and finished products. A solution procedure was developed to determine an optimal ordering policy for the procurement of raw materials and the production batch size that minimizes the total system costs. Çömez et al. [20] considered a centralized inventory sharing system of two retailers that are replenished periodically. They assumed that, between two replenishments, a unit can be transshipped to a stocked-out retailer from the other retailer. Whenever there is an absence of transshipments, the backorder costs are incurred until the next replenishment. The objective of their study is to minimize the long-run average costs, comprising the replenishment, holding, backorder, and transshipment costs. They discussed the challenges associated with positive replenishment time and developed upper and lower bounds of average costs in such situations. Other studies [21–29] focused on various aspects of periodic or multiple delivery issues in the vendor-buyer integrated supply chains.

With the purpose of addressing the real-life production-shipment situation, this study extends a recent work [9] by incorporating a multiple delivery policy into their model to replace the continuous policy and investigates the effect on the optimal run time decision for this specific EPQ model. Next, we further expand the scope of the problem to combine the retailer's stock holding cost into our study. This enhanced EPQ-based model can be used to reflect the situation found in contemporary manufacturing firms in which finished products are delivered to the producer's own retail stores and stocked there for sale. The objectives are to determine the optimal run times that minimize the expected total system costs comprising costs incurred in production units, transportation, and retail stores for both models. As little attention has been paid to this specific area, the present study is intended to fill this gap.

## 2. Statement and Optimization of Proposed Model 1

In real supply chain environments, the discontinuous multidelivery policy is often used when finished products need to be transported to retail stores or customers outside the production units. To explicitly address this realistic situation, the first proposed model in this study incorporates a multiple delivery policy into an EPQ-based inventory model with scrap items and breakdown [9] to replace the continuous issuing policy and investigates the effect on the optimal manufacturing run time decision.

**Figure 1:**
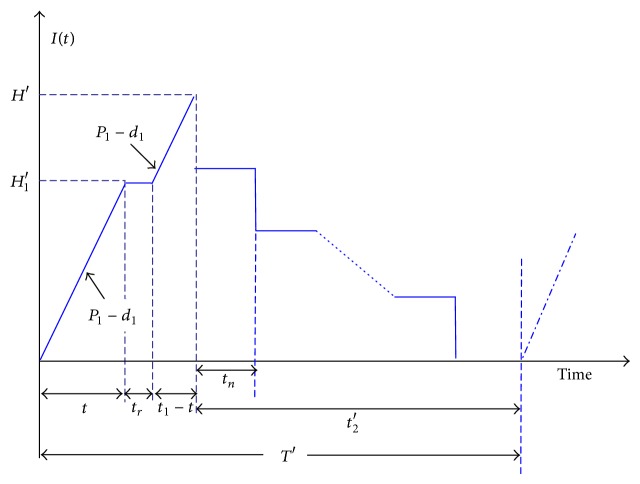
Inventory level of finished items in the proposed manufacturing run time problem.

Summary of assumptions (features) considered in the proposed multi-item EPQ-based model are as follows: (1) a random machine breakdown rate, (2) a random scrap rate in production, and (3) a discontinuous multidelivery policy for finished products. The details of the proposed model can be described as follows. Suppose a product can be manufactured at an annual rate *P*_1_ and its demand is *λ* units per year. All items produced must pass a quality conformation check, and the unit screening cost is included in the unit manufacturing cost *C*. A random *x* proportion of the products produced is defective and will be scrapped at the end of the regular production process. Hence, the production rate of scraps is *d*_1_ and *d*_1_ = *P*_1_ *x*. Under regular operations (i.e., to avoid a stock-out situation) (*P*_1_ − *d*_1_ − *λ*) > 0 must be satisfied. Upon the completion of the production process, the acceptable quality (finished) products are transported to the outside retail store or customer, under a discontinuous multidelivery policy, while fixed quantity *n* installments of the finished items are shipped to retail store at a fixed interval of time in *t*_2_′ (Figure 1). The proposed model assumes that, during the production uptime, a Poisson distributed machine breakdown may occur, and an abort/resume (A/R) inventory control policy is employed when a breakdown happens. Under such an A/R policy, the machine goes under repair immediately and a constant repair time is assumed. Upon the completion of the repair, the interrupted lot is instantly resumed (Figure 1).

Additional cost-related parameters used in this study are the machine repairing cost *M*, setup cost per cycle *K*, holding cost per item at the producer's side *h*, disposal cost per scrapped item *C*_*S*_, fixed delivery cost per shipment *K*_1_, variable delivery cost per item *C*_*T*_, unit holding cost for safety stock at the producer's side *h*_3_, and holding cost per item at the retailer's side *h*_2_. Other notations used in the modeling and analysis also include the following:

 
*t*: production time before a random machine breakdown takes place,
 
*H*_1_′: on-hand inventory level in units when a random machine breakdown takes place,
 
*β*: number of machine breakdowns per unit time (i.e., year), assumed to be a random variable that follows the Poisson distribution,
 
*t*_*r*_: machine repair time,
 
*t*_1_: production uptime, the decision variable of the proposed manufacturing run time model,
 
*H*′: maximum on-hand inventory level in units when the regular production process ends (in the case of a breakdown),
 
*t*_2_′: time required to deliver all finished items produced in a cycle (in the case of a breakdown),
 
*T*′: production cycle length (in the case of a breakdown),
 
*Q*: lot size for each production cycle,
 TC_1_(*t*_1_): total production-inventory-delivery costs per cycle (in the case of a breakdown),
 
*E*[TC_1_(*t*_1_)]: the expected production-inventory-delivery costs per cycle (in the case of a breakdown),
 
*t*_2_: time required to deliver all finished items produced (in the case of no breakdown),
 
*H*: on-hand inventory level in units when the regular production process ends (in the case of no breakdown),
 
*T*: cycle length (in the case of no breakdown),
 
*I*(*t*): on-hand inventory level of finished items at time *t*,
 
*I*_*s*_(*t*): on-hand inventory level of scrap items at time *t*,
 TC_2_(*t*_1_): total production-inventory-delivery costs per cycle (in the case of no breakdown),
 
*E*[TC_2_(*t*_1_)]: the expected production-inventory-delivery costs per cycle (in the case of no breakdown),
 
*TCU*⁡(*t*_1_): total production-inventory-delivery costs per unit time whether or not a breakdown takes place,
 
*E*[*TCU*⁡(*t*_1_)]: the long-run expected production-inventory-delivery costs per unit time whether or not a breakdown takes place,
 
**T**: the cycle length whether or not a machine breakdown takes place.

Since a machine breakdown may randomly take place at production uptime *t*_1_, the following two distinct cases must be examined.

### 2.1. Case  1: A Random Machine Breakdown Takes Place at Uptime *t*_1_

**Figure 2:**
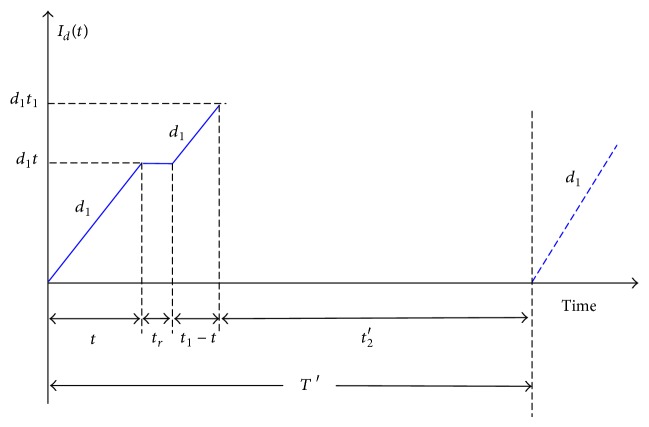
Inventory level of scrap items in the proposed manufacturing run time problem.

In such a situation, *t* < *t*_1_. Under the AR inventory control policy, the machine goes under repair immediately, and once it is fixed and restored, the interrupted lot is instantly resumed (Figure 1). Since *x* proportion of scrap products is produced, the maximum number of scraps in a cycle is *xQ* (or *d*_1_ *t*_1_), and the on-hand inventory of scrap items in the proposed manufacturing run time problem is as illustrated in Figure 2.

The production cycle time *T*′ can be seen as (1) from Figure 1

(1) $$\begin{array}{c} T^{\prime }=t_{1}+t_{r}+t_{2}^{\prime }. \end{array}$$

The total production-inventory-delivery cost per cycle, TC_1_(*t*_1_), is comprised of (1) the variable production cost, (2) the setup cost, (3) the disposal cost for scraps, (4) the machine repair cost, (5) fixed and variable product delivery costs, (6) holding cost for safety stocks, and (7) the producer's inventory holding costs in the entire production cycle. Thus, TC_1_(*t*_1_) is

(2) TC1t1=CP1t1+K+CSt1P1x+M+nK1+CTt1P1+h3(λtr)T′+hH′+d1t12t1+H1′+d1ttr+n−12nH′t2′.

Since *x* is assumed to be a random variable with a known probability density function, the expected values of *x* are used in our analysis to take the randomness of *x* into account. By substituting all related system parameters into (2) [9], with further derivations, *E*[TC_1_(*t*_1_)] becomes (see the appendix for more details)

(3) ETC1t1=K+nK1+M+htP1g +1−1nhP1g(1−Ex)2CP1+CSP1Ex+CTP11−Ex   +h3P1g(1−Ex)−hP1g(1−Ex)21−1n·t1 +hP1Ex2+hP122λ1−Ex21−1nP1x2+hP12n1−ExhP1Ex2+hP122λ1−Ex21−1nt12.

### 2.2. Case  2: No Breakdown Takes Place at Uptime *t*_1_

**Figure 3:**
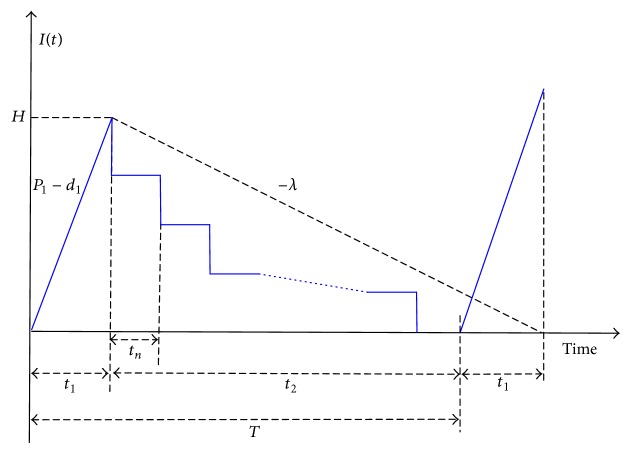
Inventory level of finished items in a manufacturing run time problem with no breakdown, defective rate, and discontinuous delivery policy.

In such a situation, *t* > *t*_1_. The inventory level of finished items in this case is depicted in Figure 3, and *T* = *t*_1_ + *t*_2_. The total production-inventory-delivery cost per cycle TC_2_(*t*_1_) is as displayed in

(4) TC2t1=CP1t1+K+CSxt1P1+nK1 +CTt1P11−x+h3(λtr)T +hH+d1t12t1+n−12nHt2.

Again, to take the randomness of *x* into account and substitute all related parameters into (4), with further derivations, *E*[TC_2_(*t*_1_)] becomes [12]

(5) ETC2t1=K+nK1+CP1+CSExP1+CTP1(1−Ex)  +h3P1g1−Ext1+hP1Ex2+hP122λ1−Ex21−1n  +hP12n1−ExhP1Ex2+hP122λ1−Ex21−1nt12.

### 2.3. Integration of the Proposed Run Time Models with/without Breakdown

A machine breakdown may take place randomly and it follows a Poisson distribution with mean equal to *β* per year. Let *f*(*t*) be the probability density function of random time *t* before a breakdown takes place, and *F*(*t*) represents the cumulative density function of *t*. Hence, the long-run expected system costs per unit time *E*[*TCU*⁡(*t*_1_)] are

(6) ETCU⁡t1 =∫0t1ETC1t1ftdt+∫t1∞ETC2t1ftdtET,

where

(7) ET=∫0t1ET′ftdt+∫t1∞ETftdt=t1P11−Exλ.

From Figures 1 and 3, it can be seen that *T*′ and *T* are different in length (*T*′ is longer than *T* since it contains machine repairing time) and because a breakdown can occur randomly, it is necessary to use the integration (i.e., equation (7)) to derive the expected cycle length.

It is also noted that the time between breakdowns obeys the exponential distribution with density function *f*(*t*) = *βe* ^−*βt*^ and cumulative density function *F*(*t*) = 1 − *e* ^−*βt*^. By substituting *E*[TC_1_(*t*_1_)], *E*[TC_2_(*t*_1_)], and *E*[**T**] into (6) and solving the integration of the mean time to breakdown in *E*[*TCU*⁡(*t*_1_)], we obtain

(8) ETCU⁡t1 =λ1−Ex  ·K+nK1t1P1+γ1+γ2t12+MP1+hgβ1−e−βt1t1  i1+−hge−βt1−hg1−Ex21−1n1−e−βt1K+nK1t1P1+γ1+γ2t12+MP1+hgβ1−e−βt1t1,

where

(9) $$\begin{array}{c} \gamma_{1}=\left[C+C_{S}E\left[x\right]+C_{T}\left(1-E\left[x\right]\right)+h_{3}g\left(1-E\left[x\right]\right)\right], \end{array}$$

(10) $$\begin{array}{c} \gamma_{2}=\left[\frac{hP_{1}}{\lambda }{\left(1-E\left[x\right]\right)}^{2}\left(1-\frac{1}{n}\right)+hE\left[x\right]+\frac{h}{n}\left(1-E\left[x\right]\right)\right]. \end{array}$$

### 2.4. Derivation of the Optimal Production Run Time

In order to derive the optimal production run time *t*_1_^*^, we first have to prove that *E*[*TCU*⁡(*t*_1_)] is convex. Let *ξ*(*t*_1_) represent the following:

(11) $$\begin{array}{c} \xi \left(t_{1}\right)=\frac{2\left(K+nK_{1}\right)\beta +2\left(1-e^{-\beta t_{1}}\right)\gamma_{4}}{\left[t_{1}^{2}P_{1}\beta^{2}\gamma_{3}+\gamma_{4}\left(2+\beta t_{1}\right)\right]\beta e^{-\beta t_{1}}}. \end{array}$$

**Theorem 1 (*E*[*TCU*⁡(*t*_1_)] is convex if 0 < *t*_1_ < *ξ*(*t*_1_)).**

The second derivative of *E*[*TCU*(*t*_1_)] with respect to *t*_1_ is

(12) d2ETCUt1d2t12=λ1−Ex ·MP1+hgβ21−e−βt1t13−2βe−βt1t12−β2e−βt1t12K+nK1t13P1−hg1−1−Ex21−1nβ2e−βt1  +MP1+hgβ21−e−βt1t13−2βe−βt1t12−β2e−βt1t1.

It is noted that because annual demand *λ* > 0, the first term in the right-hand size (RHS) of (12) is positive. Hence, we obtain

(13) d2ETCUt1dt12>0if  MP1+hgβ21−e−βt1t13−2βe−βt1t12−β2e−βt1t12K+nK1t13P1−hg1−1−Ex21−1nβ2e−βt1if +MP1+hgβ21−e−βt1t13−2βe−βt1t12−β2e−βt1t1if  >0.

The RHS of (13) can be further derived as

(14) if 1−1−E[x]21−1n2K+nK1β−t13P1βhgi   ·1−1−E[x]21−1nβ2e−βt1i   +Mβ+hgP1i   ×21−e−βt1−2t1βe−βt1−β2t12e−βt11−1−Ex21−1n    >0.

Let

(15) $$\begin{array}{c} \gamma_{3}=hg\left[1-\frac{\left(1-E\left[x\right]\right)}{2}\left(1-\frac{1}{n}\right)\right],\\ \gamma_{4}=\left(M\beta +hgP_{1}\right); \end{array}$$

then, (13) can be rewritten as

(16) d2ETCUt1dt12>0if  2K+nK1β+21−e−βt1γ4if  w−t1t12P1β2γ3+γ42+βt1βe−βt1if  w>0

or

(17) d2ETCUt1dt12>0if  0<t1<2K+nK1β+21−e−βt1γ4t12P1β2γ3+γ42+βt1βe−βt1=ξt1.

If *E*[*TCU*(*t*_1_)] is a convex function, then the minimum point exists. In order to locate the optimal production run time *t*_1_^*^ that minimizes *E*[*TCU*(*t*_1_)], we can set the first derivative of *E*[*TCU*(*t*_1_)] equal to zero and solve *t*_1_^*^:

(18) dETCUt1dt1=λ1−Ex ·MP1+hgβ−1−e−βt1t12+βe−βt1t1−K+nK1t12P+γ22+γ3βe−βt1 · +MP1+hgβ−1−e−βt1t12+βe−βt1t1.

In the RHS of (18), it can be seen that the first term is positive, so the second term is equal to zero. Let

(19) t1U∗=2γ4+Kβ+nK1βγ2P1β,

(20) t1L∗=the positive root of −γ4±γ42+2P1γ2+2βγ3K+nK1P1γ2+2βγ3.

Theorem 2 (*t*_1L_^*^ < *t*_1_^*^ < *t*_1U_^*^).

Because the proof of *t*_1_^*^ falls within the upper and lower bounds, we can multiply the second term of (18) by (2*βP*_1_ *t*_1_^2^) and obtain

(21) P1βγ2+2P1β2γ3e−βt1t12+2γ4βe−βt1t1 −2βK+nK1+γ41−e−βt1=0.

Thus

(22) t1∗=the positive root of  ×−2βK+nK1+γ41−e−βt12γ4βe−βt121/2  −2γ4βe−βt1 i ±2γ4βe−βt12 1111−4P1βγ2+2P1β2γ3e−βt1 1122211×−2βK+nK1+γ41−e−βt12γ4βe−βt121/2  ×2γ2P1β+2P1β2γ3e−βt1−1×−2βK+nK1+γ41−e−βt12γ4βe−βt121/2.

Equation (21) can be rearranged as

(23) 2P1β2γ3t12+γ4βt1+γ4e−βt1 =2βK+nK1+γ4−P1βγ2t12

or

(24) $$\begin{array}{c} e^{-\beta t_{1}}=\frac{2\left[\beta \left(K+nK_{1}\right)+\gamma_{4}\right]-\left(P_{1}\beta \gamma_{2}t_{1}^{2}\right)}{2\left[P_{1}\beta^{2}\gamma_{3}t_{1}^{2}+\gamma_{4}\beta t_{1}+\gamma_{4}\right]}, \end{array}$$

where *e* ^−*βt*_1_^ is the complement of the cumulative density function *F*(*t*_1_) = 1 − *e* ^−*βt*_1_^. As 0 ≤ *F*(*t*_1_) ≤ 1, 0 ≤ *e* ^−*βt*_1_^ ≤ 1. Let *e* ^−*βt*_1_^ = 0 and *e* ^−*βt*_1_^ = 1 be the upper and lower bounds for *e* ^−*βt*_1_^, respectively. By substituting them into (22), we obtain

(25) t1U∗=2βK+nK1+γ4γ2P1β,t1L∗=the positive root of−γ4±γ42+2P1γ2+2βγ3K+nK1P1γ2+2βγ3,

and *t*_1L_^*^ < *t*_1_^*^ < *t*_1U_^*^.

It is noted that although the optimal production run time *t*_1_^*^ cannot be presented in a closed form, it does fall within the bounds. *t*_1_^*^ can be located with the use of a proposed recursive searching algorithm. Let

(26) ωt1=e−βt1=2βK+nK1+γ4−P1βγ2t122P1β2γ3t12+γ4βt1+γ4 ∴0≤ω(t1)≤1.

In order to locate *t*_1_^*^, we can use the following recursive searching algorithm.

1. Let *ω*(*t*_1_) = 0 and *ω*(*t*_1_) = 1 initially and calculate the upper and lower bounds for *t*_1_^*^, respectively (i.e., to obtain the initial values of [*t*_1L_^*^, *t*_1U_^*^]).
2. Substitute the current values of [*t*_1L_^*^, *t*_1U_^*^] into *e* ^−*βt*_1_^ and compute the new bounds, expressed as *ω*_*L*_ and *ω*_*U*_ for *e* ^−*βt*_1_^. Hence, *ω*_*L*_ < *ω*(*t*_1_) < *ω*_*U*_.
3. Let *ω*(*t*_1_) = *ω*_*L*_ and *ω*(*t*_1_) = *ω*_*U*_ and update the upper and lower bounds for *t*_1_^*^, respectively (i.e., to obtain the new values of [*t*_1L_^*^, *t*_1U_^*^]).
4. Repeat steps (2) and (3) until there is no significant difference between *t*_1L_^*^ and *t*_1U_^*^ (or there is no significant difference in terms of their effects on *E*[*TCU*⁡(*t*_1_^*^)]).
5. Stop. *t*_1_^*^ is found.

## 3. Extension to a Producer-Retailer Integrated EPQ-Based System (Model 2)

### 3.1. Enhanced Model Description and Formulation

**Figure 4:**
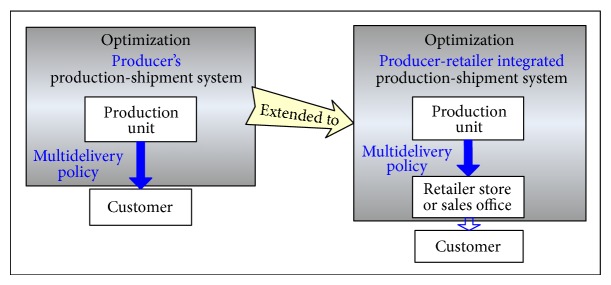
Extension to a producer-retailer integrated production-shipment system.

In this section, we further extend the scope of the problem to incorporate the retailer's stock holding cost into our study. The new model can be considered a producer-retailer integrated system, because, in the present-day manufacturing sector, some producers of consumer goods may own and operate retail stores or regional sales offices to promote and sell their end products to customers (see Figure 4). With the intention of addressing such a real-life intrasupply chain situation, the second model of this study incorporates the retailer's stock holding cost into the first model and investigates its effect on the optimal production run time decision.

**Figure 5:**
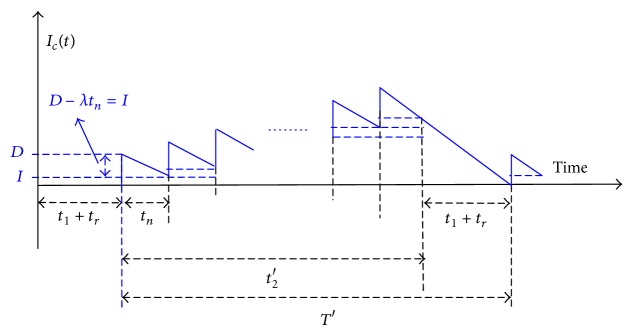
Inventory level of finished products on the retailer's side in the proposed manufacturing run time problem with breakdown.

In the proposed study, the retailer's stock holding positions are illustrated in Figure 5.

Extra parameters used in this enhanced model include the following.

 
*h*_2_: holding cost per product stored on the retailer's side,
 
*I*_*c*_(*t*): on-hand inventory levels in units on the retailer's side end at time *t*,
 
*D*: number of finished products (a fixed quantity) transported to the retail store per shipment,
 
*I*: number of left-over products in *t*_*n*_ after satisfying the demand in *t*_*n*_,
 TC_3_(*t*_1_): total production-inventory-delivery costs per cycle of this enhanced model (in the case of a breakdown),
 TC_4_(*t*_1_): total production-inventory-delivery costs per cycle of this enhanced model (in the case of no breakdown),
 
*E*[TC_3_(*t*_1_)]: the expected production-inventory-delivery costs per cycle of this enhanced model (in the case of a breakdown),
 
*E*[TC_4_(*t*_1_)]: the expected production-inventory-delivery costs per cycle of this enhanced model (in the case of no breakdown),
 
*E*[*TCU*⁡_*e*_(*t*_1_)]: the long-run expected production-inventory-delivery costs per unit time in this enhanced model, whether or not a breakdown takes place.

Since the demand on the retailer's side in time interval *t*_*n*_ is *λt*_*n*_, after satisfying the demand, the number of left-over items (see Figure 5) in each *t*_*n*_ is

(27) $$\begin{array}{c} I=D-\lambda t_{n}. \end{array}$$

Total inventory holding costs on retailer's side with and without breakdown are shown, respectively, in

(28) $$\begin{array}{c} h_{2}\left[n\frac{\left(D-I\right)}{2}t_{n}+\frac{n\left(n+1\right)}{2}It_{n}+\frac{nI}{2}\left(t_{1}+t_{r}\right)\right], \end{array}$$

(29) $$\begin{array}{c} h_{2}\left[n\frac{\left(D-I\right)}{2}t_{n}+\frac{n\left(n+1\right)}{2}It_{n}+\frac{nI}{2}\left(t_{1}\right)\right]. \end{array}$$

To incorporate the retailer's holding costs into the original models with and without breakdown, respectively, we obtain

(30) TC3t1=TC1t1 TC3t1+h2nD−I2tn+nn+12Itn+nI2t1+tr,TC4t1 =TC2t1+h2nD−I2tn+nn+12Itn+nI2t1.

To take the randomness of defective rate *x* into account and substitute all related variables into (30), with further derivations, *E*[TC_3_(*t*_1_)] and *E*[TC_4_(*t*_1_)] can be obtained as follows:

(31) ETC3t1=ETC1t1+h2P1g1−Ex2·1−1nt1+h2P11−Ex2·P11−Exλn+1−1nt12,ETC4t1=ETC2t1+h2P11−Ex2·P11−Exλn+1−1nt12.

### 3.2. Integration of Enhanced Model with/without Breakdown

The mean time to breakdowns obeys the exponential distribution with *f*(*t*) = *βe* ^−*βt*^. Therefore, *E*[*TCU*⁡_2_(*t*_1_)] is

(32) ETCU⁡2t1=∫0t1ETC3t1ftdt+∫t1∞ETC4t1ftdtET.

Substituting *E*[*TCU*⁡_3_(*t*_1_)], *E*[TC_4_(*t*_1_)], and *E*[**T**] into (32) and resolving *E*[*TCU*⁡_2_(*t*_1_)], we obtain

(33) ETCU⁡2t1=λ1−Ex ·K+nK1t1P1+γ1+γ2t12+γ5t1  +MP1+hgβ1−e−βt1t1−hge−βt1  −h−h2g1−Ex21−1n1−e−βt1K+nK1t1P1,

where

(34) $$\begin{array}{c} \gamma_{5}=\frac{h_{2}\left(1-E\left[x\right]\right)}{2}\left[\frac{P_{1}\left(1-E\left[x\right]\right)}{\lambda n}+\left(1-\frac{1}{n}\right)\right]. \end{array}$$

### 3.3. Determining the Optimal Run Time

Let *ψ*(*t*_1_) stand for the following:

(35) $$\begin{array}{c} \psi \left(t_{1}\right)=\frac{2\left(K+nK_{1}\right)\beta +2\gamma_{4}\left(1-e^{-\beta t_{1}}\right)}{\left[t_{1}^{2}P_{1}\beta^{2}\gamma_{6}+\gamma_{4}\left(2+\beta t_{1}\right)\right]\beta e^{-\beta t_{1}}}. \end{array}$$

**Theorem 3 (*E*[TCU_2_(*t*_1_)] is convex if 0 < *t*_1_ < *ψ*(*t*_1_)).**

The second derivative of *E*[*TCU*_2_(*t*_1_)] with respect to *t*_1_ is

(36) d2E[TCU2(t1)]d2t12=λ1−Ex−2βe−βt1t12−β2e−βt1t121−e−βt1t132K+nK1t13P1+h−h2g1−Ex2=eλ1−Ex ·1−1nβ2e−βt1−hgβ2e−βt1=eλ1−Ex +MP1+hgβ21−e−βt1t13=eλ1−Ex +MP1+hgβ −2βe−βt1t12−β2e−βt1t121−e−βt1t13.

Since annual demand *λ* > 0, the first term in the RHS of (36) is positive, and

(37) if  21−e−βt1t13−2βe−βt1t12−β2e−βt1t12K+nK1t13P1+h−h2g1−Ex21−1n  ·β2e−βt1−hgβ2e−βt1+MP1+hgβ  ·21−e−βt1t13−2βe−βt1t12−β2e−βt1t1>0then  d2ETCU2t1dt12>0.

With further derivations, the left-hand side (LHS) of (37) becomes

(38) if  2K+nK1β−t13P1βh−h2g1−Ex21−1n  ·β2e−βt1−t13P1βhgβ2e−βt1  +Mβ+hgP121−e−βt1−2t1βe−βt1−β2t12e−βt1g1−Ex21−1n  >0.

Let

(39) $$\begin{array}{c} \gamma_{6}=\left\{\left(h-h_{2}\right)\left[\frac{g\left(1-E\left[x\right]\right)}{2}\left(1-\frac{1}{n}\right)\right]+hg\right\}; \end{array}$$

then, (37) becomes

(40) if  2K+nK1β+2γ41−e−βt1  −t1t12P1β2γ6+γ42+βt1βe−βt1>0then  d2ETCU2t1dt12>0

or

(41) d2ETCU2t1dt12>0if  0<t1<2K+nK1β+2γ41−e−βt1t12P1β2γ6+γ42+βt1βe−βt1=ψt1.

Once *E*[*TCU*_2_(*t*_1_)] is proven to be convex, the optimal run time *t*_1_^*^ can be solved by setting the first derivative of *E*[*TCU*_2_(*t*_1_)] = 0:

(42) dETCU2t1dt1=λ1−Ex·MP1+hgβ−1−e−βt1t12+βe−βt1t1−K+nK1t12P+γ22+γ5+γ6βe−βt1=eλ1−Ex· +MP1+hgβ−1−e−βt1t12+βe−βt1t1=0.

It can be seen that the first term in the RHS of (42) is positive, so the second term is equal to zero. In order to find the bounds for *t*_1_^*^, let

(43) t1U∗=2βK+nK1+γ4P1βγ2+2γ5,

(44) t1L∗=the positive root of−γ4±γ42+2P1K+nK1γ2+2γ5+2γ6βP1γ2+2γ5+2γ6β.

Theorem 4 (*t*_1L_^*^ < *t*_1_^*^ < *t*_1U_^*^).

For the proof of Theorem 4 please refer to the proof for Theorem 2 in Section 2.

Once we are certain that *t*_1_^*^ falls within the aforementioned upper and lower bounds, in order to find *t*_1_^*^, we can first multiply the second term of (42) by (2*P*_1_ *t*_1_^2^ *β*) and obtain the following:

(45) P1βγ2+2γ5+2P1β2γ6e−βt1t12+2γ4βe−βt1t1 −2βK+nK1+γ41−e−βt1 =0.

Equation (45) can be rearranged as

(46) $$\begin{array}{c} e^{-\beta t_{1}}=\frac{2\left[\beta \left(K+nK_{1}\right)+\gamma_{4}\right]-\left[P_{1}\beta \left(\gamma_{2}+2\gamma_{5}\right)\right]t_{1}^{2}}{2\left[P_{1}\beta^{2}\gamma_{6}t_{1}^{2}+\gamma_{4}\left(1+\beta t_{1}\right)\right]}, \end{array}$$

where *e* ^−*βt*_1_^ is the complement of the cumulative density function *F*(*t*_1_) = 1 − *e* ^−*βt*_1_^. As 0 ≤ *F*(*t*_1_) ≤ 1, 0 ≤ *e* ^−*βt*_1_^ ≤ 1. Let *e* ^−*βt*_1_^ = 0 and *e* ^−*βt*_1_^ = 1 be the initial upper and lower bounds of *e* ^−*βt*_1_^, respectively. Then, by using the proposed recursive searching algorithm given at the end of Section 2, we can find the optimal production run time *t*_1_^*^.

## 4. Numerical Example

In order to relieve the comparison efforts for readers, this section adopts the same numerical example as in [9]. For a demonstration of the proposed EPQ-based model 1, the following system parameters are used.

*P*_1_: production rate, 10,000 products per year;
*λ*: demand rate, 4,000 products per year;
*x*: random scrap rate, which follows uniformly distribution over the interval [0, 0.2];
*β*: Poisson breakdown rate, 0.5 average times per year;
*g*: constant machine repair time *t*_*r*_, 0.018 year per repair;
*M*: machine repair cost, $500 for each breakdown;
*K*: setup cost, $450 per production run;
*C*: manufacturing cost, $2 per item;
*C*_*S*_: disposal cost, $0.3 per scrap item;
*h*: holding cost, $0.6 per item per unit time;
*K*_1_: fixed delivery cost, $90 per shipment;
*n*: number of deliveries, 4 per cycle;
*C*_*T*_: variable delivery cost, $0.001 per item.

**Figure 6:**
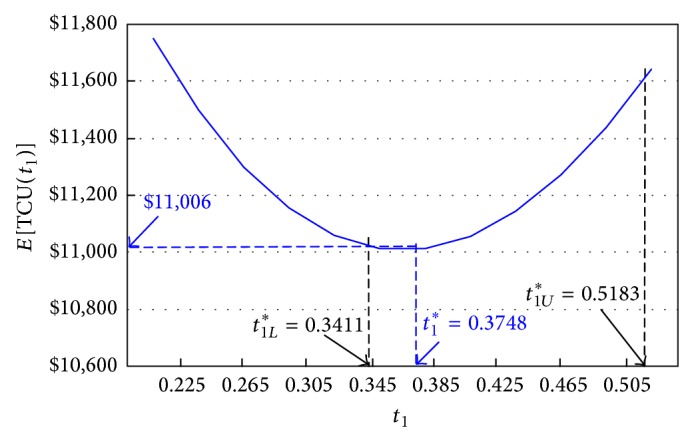
The behavior of *E*[*TCU*⁡(*t*_1_)] in connection with the production run time *t*_1_ in the proposed model 1.

First, we use both upper and lower bounds of *t*_1_^*^ to test for the convexity of *E*[*TCU*⁡(*t*_1_)] (see Theorem 1). The computation results of (19), (20), and (11) indicate that *t*_1U_^*^ = 0.5183 < *ξ*(*t*_1U_^*^) = 2.8723, and *t*_1L_^*^ = 0.3411 < *ξ*(*t*_1L_^*^) = 2.6261. Hence, [*TCU*⁡(*t*_1_)] is convex (Figure 6).

In order to find the optimal *t*_1_^*^, we first substitute the upper and lower bounds of *t*_1_^*^ in (8) and obtain *E*[*TCU*⁡(*t*_1U_^*^)] = $11,601.63 and *E*[*TCU*⁡(*t*_1L_^*^)] = $11,014.50, respectively. Because the optimal run time *t*_1_^*^ falls within the interval of [*t*_1L_^*^, *t*_1U_^*^], we apply the proposed recursive searching algorithm stated at the end of Section 2 and find *t*_1_^*^ = 0.3748 years. Accordingly, the optimal expected system costs per unit time *E*[*TCU*⁡(*t*_1_^*^)] = $11,006.41 (Figure 6).  Table 1 shows the step-by-step iterations of the algorithm.

In this specific studied model, we focus on incorporating a discontinuous multidelivery policy into a prior work [9] and consider a fixed transportation cost associated with each delivery. Applying the research result, we can easily investigate the effects of different fixed transportation costs *K*_1_ on the optimal system cost *E*[*TCU*⁡(*t*_1_^*^)] and on the optimal production run time *t*_1_^*^ (see Table 2). It can be seen from Table 2 that as the ratio of *K*_1_/*K* increases, the expected system costs per unit time *E*[*TCU*⁡(*t*_1L_^*^)] increase significantly. It is also noted that as *K*_1_ increases, optimal production run time *t*_1_^*^ also increases significantly.

### 4.1. Numerical Example for the Producer-Retailer Integrated EPQ System (Model 2)

In order to demonstrate the research result of the producer-retailer integrated EPQ-based model, an additional system variable *h*_2_ = $1.50 per item stored at the retailer's side is included.

Again, one can use the upper and lower bounds of *t*_1_^*^ (equations (43) and (44)) to test for convexity of *E*[*TCU*⁡(*t*_1_)] (Theorem 3 and equation (35)). The results reveal that *t*_1U_^*^ = 0.3213 < *ψ*(*t*_1U_^*^) = 2.6462 and *t*_1L_^*^ = 0.2186 < *ψ*(*t*_1L_^*^) = 2.4876. Therefore, the expected cost [*TCU*⁡(*t*_1_)] is convex.

Next, by applying the proposed recursive searching algorithm we can calculate that the optimal run time *t*_1_^*^ = 0.2314 years and the optimal *E*[*TCU*⁡(*t*_1_^*^)] = $12,138.49. It is noted that the computation time for reaching the optimal *t*_1_^*^ solution is 2.1 seconds (using Excel software in a desktop computer: Intel CPU G850 with 2.94 GB RAM and 2.89 GHz).

**Figure 7:**
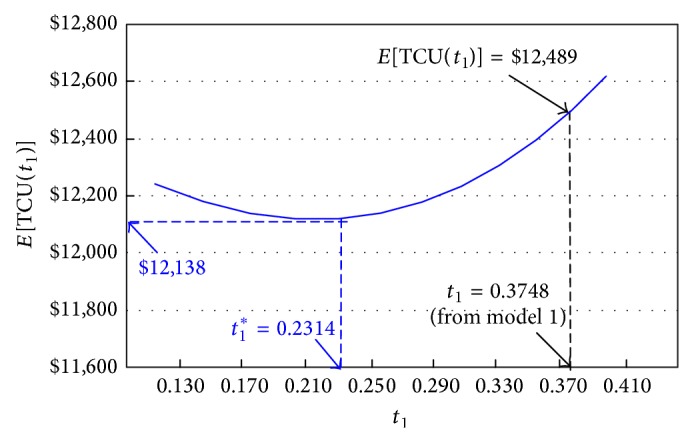
The behavior of *E*[*TCU*⁡(*t*_1_)] with respect to production run time *t*_1_ in the proposed model 2.

Figure 7 illustrates the behavior of *E*[*TCU*⁡(*t*_1_)] with regard to production run time. It is noted that, without the research result from the second model, the management of such a producer-retailer integrated system would probably use *t*_1_ = 0.3748 years (from the result of model 1) for their run time decision. Further analysis (see Figure 7) shows cost savings of $351 (or 2.9% over the total system costs) simply by applying our research result.

The effects of the unit retailer's holding cost *h*_2_ on the expected system cost *E*[*TCU*⁡(*t*_1_^*^)] and on the optimal run time *t*_1_^*^ are shown in Table 3, respectively.

It can be seen that as *h*_2_ or the ratio of *h*_2_/*h* increases, the expected cost *E*[*TCU*⁡(*t*_1_^*^)] increases, but the optimal production run time *t*_1_^*^ decreases. In decision-making, these sensitivity analyses results can provide the management of a producer-retailer integrated system with valuable information and insights into the effects of various stock holding costs in different retailers' locations.

## 5. Concluding Remarks

Two exact models for an extended EPQ-based problem with a discontinuous delivery policy, scrap rate, and random breakdown are developed in this study. They specifically address different real-life situations in production, end-item delivery, and intrasupply chains such as a producer-retailer integrated system. Mathematical modeling along with optimization techniques is used to determine the optimal production run times that minimize the expected system costs per unit time. Without in-depth investigations on these separate models, the optimal production run time and other important information related to the system parameters cannot be revealed. The proposed real-life EPQ models with* random machine breakdown*,* discontinuous product distribution policies*, and* quality assurance *must be specifically studied in order to (1) obtain the joint effects of breakdown, discontinuous distribution policies, and quality assurance on the optimal production run time; (2) get to know the effects of different policy and scope of supply chains management on the optimal run time and overall system costs; and (3) gain the insight with regard to various system's parameters of all particular EPQ-based models. Since little attention has been paid to the investigation of joint effects of these practical production situations on the optimal run time, this research is intended to bridge the gap. An interesting area for future study is the examination of the effect of variable production rates on these models.

## Figures and Tables

**Table 1:** Iterations of the recursive searching algorithm for locating t_1_^*^.

*β*	Step #	*t*_1U_^*^	*ω*_*U*_ = *e* ^−*βt*_1U_^	*t*_1L_^*^	*ω*_*L*_ = *e* ^−*βt*_1*L*_^	Difference between *t*_1U_^*^ and *t*_1L_^*^	[*U*] *E*[*TCU*⁡(t_1U_^*^)]	[*L*] *E*[*TCU*⁡(*t*_1L_^*^)]	Difference between [*U*] and [*L*]
0.5	Initial		0.00000		1.00000		$11,601.63	$11,014.50	$587.13
	1st	0.5183	0.7717	0.3411	0.8432	0.1772	$11,103.30	$11,014.50	$88.80
	2nd	0.3857	0.8246	0.3721	0.8302	0.0136	$11,007.16	$11,006.46	$0.70
	3rd	0.3756	0.8287	0.3746	0.8292	0.0010	$11,006.42	$11,006.41	$0.01
	4th	0.3749	0.8291	0.3748	0.8291	0.0001	$11,006.41	$11,006.41	$0.004
	5th	**0.3748**	**0.8291**	**0.3748**	**0.8291**	**0.00000**	**$11,006.41**	**$11,006.41**	**$0.000**

**Table 2:** Variations of the fixed delivery cost *K*_1_ effects on the optimal production run time t_1_^*^.

*K*_1_/*K*	0.05	0.2	0.4	0.6	0.8	1	1.2	1.4	1.6	1.8	2	2.2
*K*_1_	$22.5	$90	$180	$270	$360	$450	$540	$630	$720	$810	$900	$990
*E*[*TCU*⁡(t_1_^*^)]	$10,721	$11,067	$11,448	$11,773	$12,062	$12,325	$12,567	$12,793	$13,006	$13,208	$13,399	$13,583
t_1_^*^	0.3116	0.3816	0.4586	0.5244	0.5828	0.6359	0.6848	0.7305	0.7735	0.8142	0.8529	0.8900

**Table 3:** Variations of the unit retailer's holding cost *h*_2_ and their effects on *E*[*TCU*⁡(*t*_1_^*^)].

*h*_2_/*h*	0.5	0.75	1	1.25	1.5	1.75	2	2.25	**2.5**	2.75	3	3.25
*h*_2_	0.3	0.45	0.6	0.75	0.9	1.05	1.2	1.35	**1.5**	1.65	1.8	1.95
*E*[*TCU*⁡(*t*_1_^*^)]	$11,282	$11,407	$11,526	$11,638	$11,746	$11,850	$11,949	$12,045	**$12,138**	$12,229	$12,316	$12,401
*t*_1_^*^	0.3256	0.3074	0.2918	0.2785	0.2668	0.2564	0.2472	0.2389	**0.2314**	0.2246	0.2183	0.2125

## Appendix

Derivations of (3) are as follows.

Recall (2) as follows:

(A.1) TC1t1=CP1t1+K+CSt1P1x+M+nK1+CTt1P1+h3(λtr)T′+hH′+d1t12t1+H1′+d1ttr+n−12nH′t2′.

Substituting all related system parameters into (2) (please refer to the basic formulations and solution process in [9]), the TC_1_(*t*_1_) can be obtained as

(A.2) TC1t1=K+M +CP1+CSP1x+CTP11−x+h3P1g1−xt1 +nK1+hpgt−hP1g1−x2−hP1g1−x2nt1 +t12hP12+hP122λ1−x2−hP121−x+ t12w−hP122λn1−x2+hP12n1−x.

To take the randomness of *x* into account by using the expected values of *x*, with further derivations, *E*[TC_1_(*t*_1_)] can be derived as follows (i.e., equation (3)):

(A.3) ETC1t1=K+nK1+M+htP1g +hP1g1−Ex21−1nCP1+CSP1Ex+CTP1(1−Ex) +e+h3P1g1−Ex−hP1g1−Ex21−1n·t1 +hP1Ex2+hP122λ1−Ex21−1n +w+hP12n1−ExhP1Ex2+hP122λ1−Ex21−1nt12.
